# Contemporary Applications of Ultrasound in Abdominal Aortic Aneurysm Management

**DOI:** 10.3389/fsurg.2016.00029

**Published:** 2016-05-27

**Authors:** Mark Scaife, Triantafillos Giannakopoulos, Georges E. Al-Khoury, Rabih A. Chaer, Efthymios D. Avgerinos

**Affiliations:** ^1^Division of Vascular Surgery, University of Pittsburgh Medical Center, Pittsburgh, PA, USA; ^2^Department of Vascular Surgery, Athens Naval & Veterans Hospital, Athens, Greece

**Keywords:** abdominal aortic aneurysm, ultrasound, contrast-enhanced ultrasound, screening, EVAR, endoleak

## Abstract

Ultrasound (US) is a well-established screening tool for detection of abdominal aortic aneurysms (AAAs) and is currently recommended not only for those with a relevant family history but also for all men and high-risk women older than 65 years of age. The advent of minimally invasive endovascular techniques in the treatment of AAAs [endovascular aneurysm repair (EVAR)] has increased the need for repeat imaging, especially in the postoperative period. Nevertheless, preoperative planning, intraoperative execution, and postoperative surveillance all mandate accurate imaging. While computed tomographic angiography and angiography have dominated the field, repeatedly exposing patients to the deleterious effects of cumulative radiation and intravenous nephrotoxic contrast, US technology has significantly evolved over the past decade. In addition to standard color duplex US, 2D, 3D, or 4D contrast-enhanced US modalities are revolutionizing AAA management and postoperative surveillance. This technology can accurately measure AAA diameter and volume, and most importantly, it can detect endoleaks post-EVAR with high sensitivity and specificity. 4D contrast-enhanced US can even provide hemodynamic information about the branch vessels following fenestrated EVARs. The need for experienced US operators and accredited vascular labs is mandatory to guarantee the reliability of the results. This review article presents a comprehensive overview of the literature on the state-of-art US imaging in AAA management, including post-EVAR follow-up, techniques, and diagnostic accuracy.

## Introduction

Abdominal aortic aneurysm (AAA) currently is increasingly recognized as a significant cause of sudden death. AAAs are usually asymptomatic, and rupture has a mortality rate between 70 and 90% and is roughly 40% for those who survive to undergo surgery (1). Its incidence closely follows atherosclerosis and hypertension in cardiovascular mortality with just under 10,000 annual deaths in the United States attributed to aortic aneurysm or dissection (2). Population adjusted mortality is higher in the UK with over 5,500 annual deaths (3). Differences in reporting standards and prevalence of risk factors, most notably smoking, are likely the major contributors to the disparity.

Currently, measurement of aneurysm diameter is the clinically approved tool for their diagnosis, and a cutoff of 5.5 cm is used as threshold for intervention, be it open or endovascular. The advent of endovascular technology revolutionized AAA repair, decreasing perioperative morbidity and mortality, but this came at the cost of long-term complications and the need for life-long imaging surveillance. Patients are subjected to repeated exposures to X-rays: before [pre-operative computed tomographic angiography (CTA)], during (perioperative fluoroscopy and angiography), and after the procedure (postoperative and follow-up CTA). This continuous radiation exposure has raised concerns both for patients and vascular surgical teams (4–6). Additionally, the intravenous iodinated contrast required for CTAs can have deleterious effects on patients who at baseline have an impaired renal function (7).

Ultrasonography has been well established for AAA screening as it is fast, cost-effective, and free of radiation exposure as well as side effects; it is increasingly entering the field of perioperative and postoperative AAA care. Ultrasound (US) technology has significantly evolved over the past decade. In addition to standard color duplex ultrasonography (CDU), 3D, 4D, and contrast enhancement are revolutionizing AAA management and postoperative surveillance. This new technology can accurately measure not only AAA diameter but also volume, wall stress, and hemodynamic parameters, and is effective at detecting endoleaks after endovascular aneurysm repair (EVAR) (6, 8–11). This review article presents a comprehensive overview and analysis of the literature on the state-of-art US imaging in AAA management, including EVAR follow-up, techniques, and diagnostic accuracy.

## Novel Ultrasound Technologies

Ultrasonography of the aorta is primarily performed to detect or exclude the presence of a AAA. Conventional CDU is the cornerstone of screening and simple visualization of target vessels. It is cheap and readily available, and poses no risk to the patient. Limitations of US technology include its limited ability to accurately visualize the aortic aneurysm or a stent in patients who are very obese, have extensive wall calcification, subcutaneous emphysema, significant bowel gas, ascites, or a large ventral hernia (11). The resolution of the image can be improved when patients follow a low residual diet the day before and fast on the day of testing (12). While commonplace usage of CDU continues, newer technologies have vastly furthered the reach of US technology with sensitivities and specificities of some of the newer imaging modalities matching or exceeding those of CTA and MR angiography (MRA) (13).

Contrast enhancement has vastly improved the resolving capabilities of US. US contrast agents are gas-filled microbubbles that are injected into the blood stream serving as intravascular reflectors of US waves. First-generation contrast enhancement involved the use of CO_2_ bubbles as a contrast medium, but this has been wholly supplanted by sulfur hexafluoride gas with a phospholipid shell which, as it degrades, is cleared by the lungs (6). This contrast is safe for patients with impaired renal function and requires no testing or monitoring prior to administration (6). Contrast-enhanced ultrasound (CEUS) allows improved visualization of the aortic lumen and delineation of thrombotic material on the arterial wall in real time. As contrast enhancement improves resolution and it is capable to visualize the endograft from different angles demonstrating flow direction and velocity, CEUS is evolving as an attractive alternative for EVAR surveillance (see EVAR Surveillance).

Three-dimensional ultrasound (3D US) technology has recently emerged as an alternative to CTA technology. The principle of 3D reconstruction allows measurement of the maximum diameter perpendicular to the centerline of the AAA. 3D acquisition consists of an electronic sweep acquiring multiple images simultaneously in both longitudinal and transverse directions, with the transducer in a stable firm position and the patient holding his/her breath. The multiple planes are collected simultaneously, obviating the need for manual rotation of the probe with the associated possible loss of the target area of interest (11). The images that are obtained are processed with a 3D-specific software and can be enlarged, rotated, and analyzed in coronal, sagittal, and transverse planes akin to CT images (11). Speckle-tracking imaging is a post-processing method to further analyze data. The image processing algorithm automatically subdivides the region of interest into numerous small overlapping cubes with a volume of approximately 1 cm^3^. Every cube incorporates specific 3D gray scale image data, and cube displacement is tracked during the heart cycle (14). Four-dimensional ultrasound (4D US) is a term used to highlight the fact that this imaging modality provides a real-time moving 3D image of the area of interest. A 3D system can be added to many conventional probes and may also be combined with CEUS to reproduce a high-contrast 3D image. The use of non-nephrotoxic contrast media in conjunction with 3D probe guidance and reconstruction allows for real-time interrogation from any angle with sensitivity and specificity matching those of CTA and MRA (11, 13).

Strain field mapping is a novel technology that aims to analyze localized wall strain. Traditional strain mapping, or wall stress analysis, of AAAs is based on CT and MRI. US has significant advantages in both radiation exposure, cost, and, in the case of MRI, accessibility and patient tolerance. Approaches on how a model of wall strain is quantified vary. Enhancement with contrast in combination with post-processing analysis can yield a highly spatially resolved strain field (14). Other approaches use real-time three-dimensional (3D) speckle-tracking US to non-invasively describe individual wall motion (kinematics) of the infrarenal aorta with a high spatial and temporal resolution (8). This allows the strain on an area of interest to be compared to the mean as a local strain ratio (15). US has been found to have good correspondence both with prior USs and to correlate well with CT and MRI findings (10). While not yet equivalent, it is hoped that refinement of the algorithms will further increase the spatial resolution of US technology (10). The significance of this technology has yet to be fully elucidated, but the aim is to eventually correlate the values with rupture risk.

## Screening

For ease of use, low cost, and lack of radiation exposure, sonography is the well-validated highly sensitive (94–100%) and specific (98–100%) testing modality of choice for screening AAAs (16, 17). Evidence supports the use of anteroposterior rather than transverse measurements. Conventional US diameter measurement is reliable from clearly insonated parts of the aneurysm wall, which explains why the anteroposterior axis is better; transverse measurements have otherwise worse reproducibility (18). Both the external and the internal diameter may be measured (see Need for Accredited Labs). The evidence for upper threshold for AAA surveillance (5.5-cm diameter) was based on the measurement of external aortic diameter, but screening trials have also used the internal diameter (19, 20). 3D US is emerging as an even more accurate modality as it can estimate the AAA diameter perpendicular to the centerline as well as the AAA volume (21).

While there is no study demonstrating a significant difference in all-cause mortality between screened and unscreened men, there is evidence of a reduction in AAA-specific mortality by incorporating one-time ultrasonography screening among men aged 65–75 years who have ever smoked. There is no clear evidence to support general screening in women, as their low prevalence of AAA and worse outcomes associated with surgical intervention appear to outweigh any benefit. Controversy remains regarding AAA screening for men who have never smoked, women who have smoked, or have other significant risk factors for AAA. Current societal guidelines strongly recommend a screening US for male smokers 65–75 years, males with family history >50 years, while the recommendation for females is weaker, and it is only for those >65 years with multiple risk factors, smokers, or with a family history (19, 20, 22–28).

## Wall Stress Analysis and Risk Assessment

Current guidelines for repair center solely around aneurysm size, for which CDU or simple 2D ultrasonography are the cornerstone of most population-based screening programs; however, it makes intuitive sense that local wall abnormalities may prove to be independent contributors to the risk of rupture. Subjects with aneurysm diameters well under 5.0 cm can also rupture, whereas large aneurysms (> 10-cm diameter) may very well remain intact until death from other causes, indicating that the nature of the aneurysm wall may be more important than its size. Wall stress analysis has been introduced to predict the potential rupture risk of the AAA wall and is mostly performed using CT and scarcely by MRI. 3D US imaging overcomes the disadvantages of CT (radiation and contrast) and enables the possibility to acquire the vessel’s motion during the cardiac cycle. Coupled with post-collection US speckle software analysis to delineate local foci of wall abnormalities and motion deformations, wall finite element models can be calibrated to the vessel motion, and thereby, more patient-specific material properties can be derived (8, 10, 14). While only the subject of small studies and, thus far, lacking in studies of reproducibility and clinical significance, this novel technology has the power to allow for a high-resolution mapping of wall motion and strain abnormalities within an aneurysm. The drawbacks of 3D US are the low contrast compared to CT, which can be resolved using CEUS, and the limited field-of-view (FOV), which may otherwise be of minor importance (8, 10, 14, 29).

Hemodynamic variables have been also linked with aneurysm growth and rupture. Liu et al. developed the so-called echo particle image velocimetry (PIV) (30). They combined US with microbubbles acting as flow tracers in several cardiovascular models, including an AAA model. The authors managed to accurately measure several different complex flow patterns, such as vorticity, stagnation, and recirculation. Zhang et al. performed a preliminary *in vivo* study using this technique in five human carotid arteries (31). Optical PIV is currently the gold standard for wall shear rate (WSR) and wall shear stress (WSS) measurement.

It is foreseeable that this technology may permit future further stratification of AAA risk and serve as an important additional determinant of the need for intervention in addition to the presently used metrics. It is hoped that combining 3D speckle tracking with finite element analysis may permit the detection of local aortic wall abnormalities that either precede aneurysm formation or portend such to rupture.

## Pre- and Intraoperative Use of Ultrasound for EVAR

Computed tomographic angiography remains the modality of choice for accurate pre-operative planning. 3D reconstruction and consistency of measurements are as of yet unlikely to be replaced by the use of ultrasonography. Intraoperative use of CEUS has, however, been suggested as a means of reducing nephrotoxic contrast in patients with impaired renal function (32, 33). While CO_2_ angiography has been widely used in this subset of patients for arterial navigation and endograft deployment, it is generally inadequate for completion imaging (11, 32, 33).

**Figure 1:**
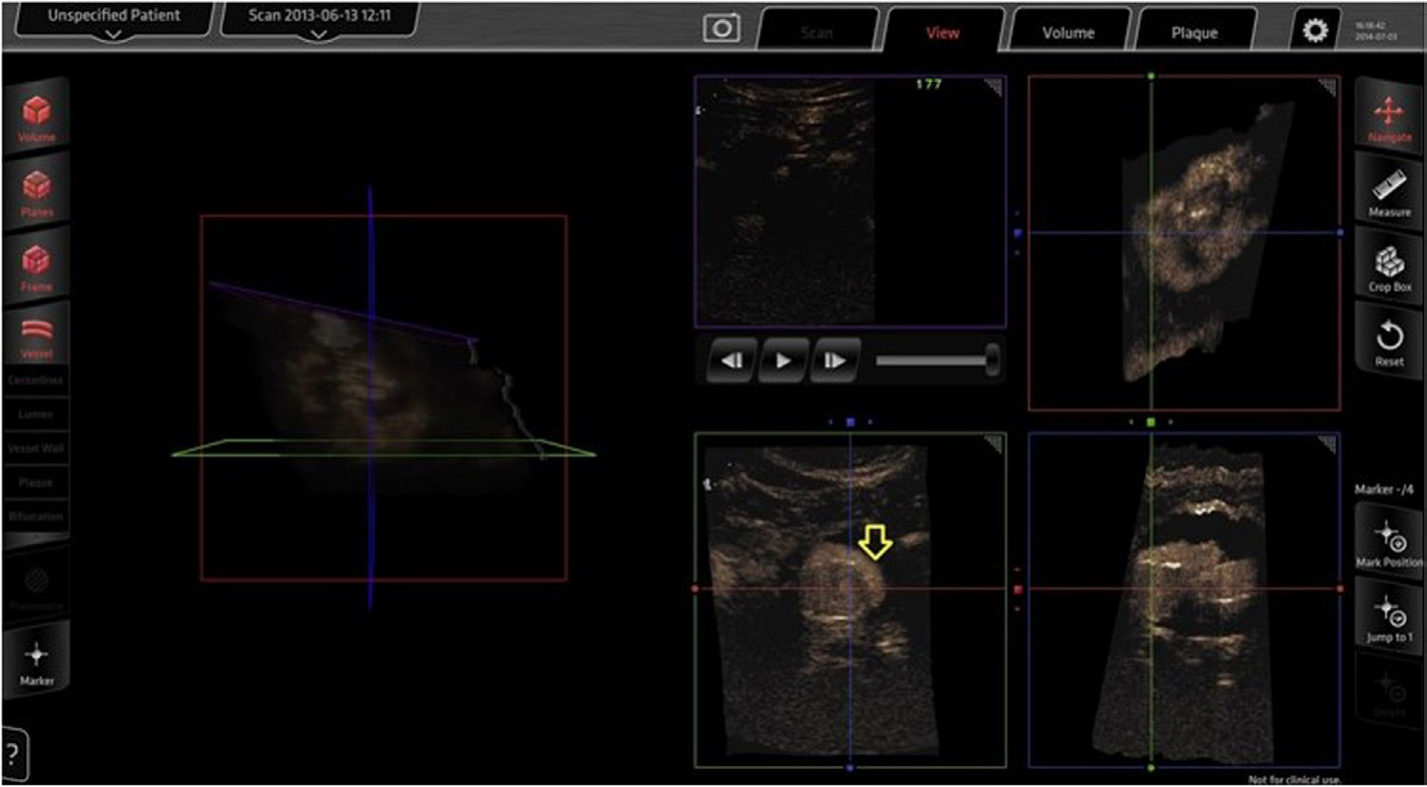
**Three-dimensional contrast-enhanced ultrasound done intraoperatively for completion imaging after EVAR**. A type I endoleak (arrow) as seen on the Curefab CS system workstation (Curefab, Munich, Germany) that was not identified on uniplanar angiography. Reprinted from Ormesher et al. (11), Copyright 2014, with permission from Elsevier.

A recent study suggested that intraoperative 3D CEUS imaging can accurately identify the renal arteries and endoleaks immediately after stent graft deployment. Furthermore, 3D CEUS imaging may detect and characterize endoleaks not seen on uniplanar angiography, including clinically important type I endoleaks, and has advantages over 2D CEUS imaging in inflow vessel identification and image manipulation (11) (Figure 1). The application uses magnetic field emitters to precisely position the US probe and interrogate an endograft from any angle within the aneurysm. Simultaneous multiplanar data acquisition allows for rapid image acquisition and subsequent high-resolution reconstruction of the target vessel. When combined with the use of CO_2_, 3D CEUS can provide satisfactory completion imaging and reduce the exposure to both radiation and nephrotoxic contrast during EVAR (11).

## EVAR Surveillance

As the durability of open aneurysm repair is well established, surveillance after the 1-month follow-up is only recommended in regular 5-year intervals to exclude a paraanastomotic aneurysm. This is typically done using CDU (27, 28).

Post-EVAR surveillance recommendations, however, have undergone significant updates, based on the relatively higher anticipated long-term complication and reintervention rates. Original practice guidelines included a postoperative 30-day CTA study, repeated at 6 months, 1 year, and annually thereafter. There is increasing evidence of the need to decrease the imaging frequency. Specifically, elimination of the 6-month follow-up study, and substitution of the CTA beyond, or even at, 1 year with CDU has been suggested (34). The follow-up protocol remains ill defined when a type II endoleak is diagnosed. Although current guidelines suggest CTA at 6 months upon type II endoleak detection at the postoperative CTA study, accumulating evidence suggests that omission of this follow-up visit and repeated imaging at 12 months with CTA or CDU (combined with radiographs to assess migration or fracture) or non-contrast-enhanced computed tomography to check for sac growth with subsequent annual CDU is adequate, provided the sac does not expand (34).

**Figure 2:**
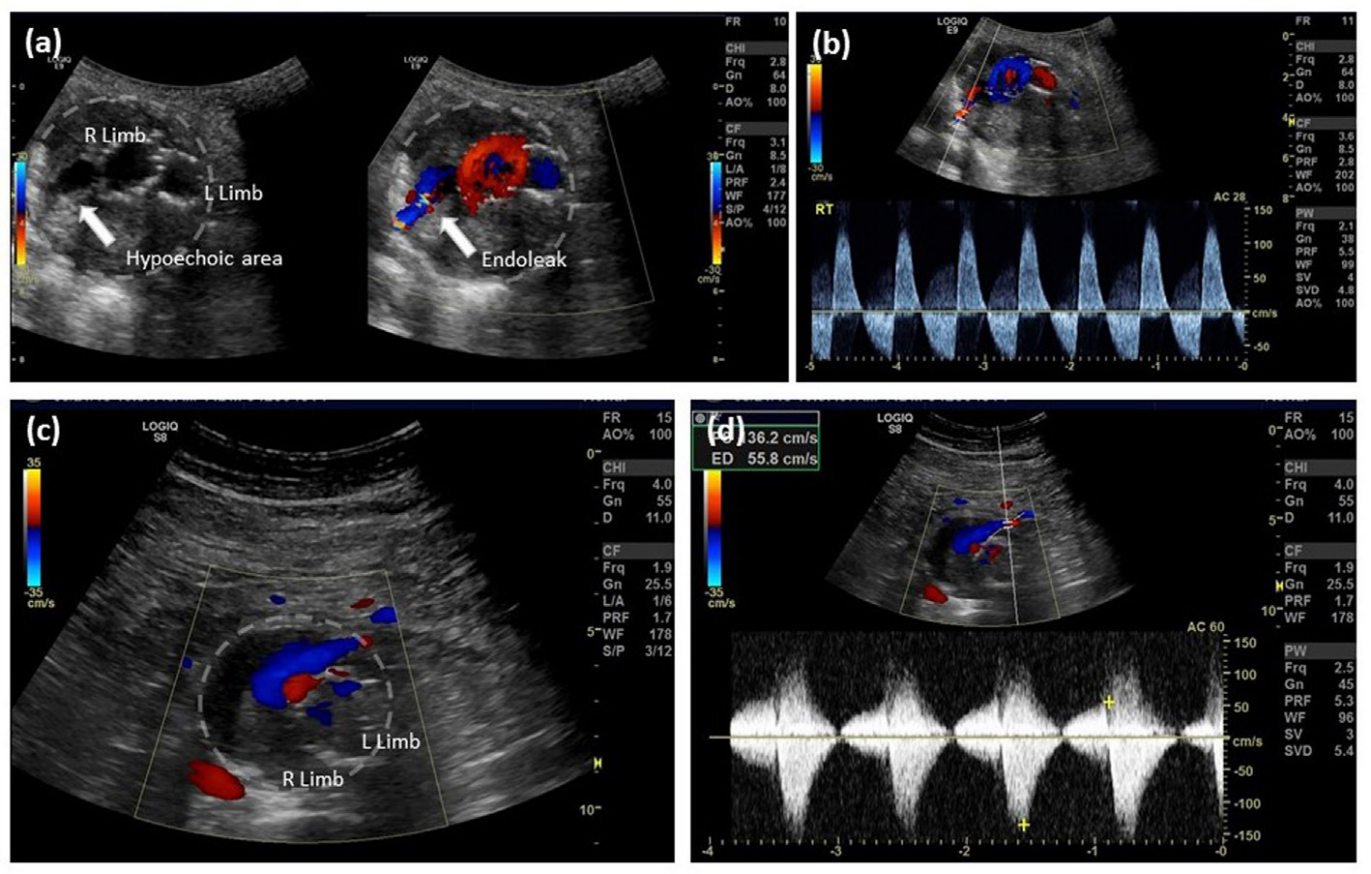
**EVAR follow-up, type II endoleak detection by color Doppler ultrasound**. **(A)** Aortic cross-sectional view showing right and left limb with a hypoechoic right posterior channel within the aortic thrombus, color Doppler filling of this channel confirms the presence of an endoleak; **(B)** spectral analysis indicates bidirectional flow along the right posterior margin of the aorta consistent with a type II endoleak from a patent lumbar artery; **(C)** aortic cross-sectional view showing right and left limb and an anterior flow channel; and **(D)** spectral analysis indicates bidirectional flow at the anterior margin of the aorta consistent with a type II endoleak from the inferior mesenteric artery.

Color duplex ultrasonography is not only cheaper and safer than other modalities but also may actually be more sensitive in the diagnosis of endoleaks, although the latter assertion remains controversial (12). Detection of type II endoleaks can be challenging and, not infrequently, they can be missed (low flow) or may overlap with other types of endoleaks and cannot be differentiated by CTA. Duplex US allows an extended period of observation; thus, it has the specific advantage of detecting not only low flow but also flow direction and characterizing the type of endoleak (Figure 2). Doppler waveforms may even predict the natural history of a type II endoleak. Color Doppler US has a reported sensitivity of 62–100% and specificity of 90–97% (12, 35, 36). CDU is capable of detecting both Type I and III endoleaks (37), and follow-up protocols based on abdominal radiography and CDU have been demonstrated to be feasible, safe, and associated with substantial annual cost savings (35, 38–40).

Contrast-enhanced ultrasound is an evolving technology that significantly increases the diagnostic resolution of ultrasonography and its ability to diagnose and detect endoleaks (41–45). Additionally, the spatial tracking and 3D reconstruction afforded by a 3D system allows for greater reproducibility and decreases operator dependency and intraobserver variability as well as provides a greater field of resolution (11, 34). CEUS has been found equivalent to CTA in endoleak detection in a number of studies with many authors going so far as to advocate the replacement of CTA with CEUS in the post-procedural surveillance of an aneurysm and detection of endoleaks (34, 38, 41–43). In the postoperative evaluation of novel grafts, such as fenestrated endovascular aneurysm grafts, 4D CEUS was found equivalent to CTA in assessing diameter, volume, endoleaks, as well as revascularized vessel patency (9). In those instances where traditional follow-up imaging modalities have failed to resolve the issue, CEUS provides an important adjunct (46, 47). It has the capacity to define a previously unclassified type of endoleak (34), visualize endoleak not previously visualized (46), and confirm vessel patency following fenestrated repairs. While the technology carries some inherent limitations which cannot be overcome, the combination of low price, no nephrotoxic contrast media or radiation exposure, reproducibility, and diagnostic accuracy equal to or greater than the current gold standard (CTA), CEUS will likely replace routine CTA as the first-line post-EVAR surveillance modality.

## Need for Accredited Labs

Ultrasound use is largely operator and equipment dependent. If the technique is not reproducible, it will never enter clinical practice. This makes standardization of results and especially AAA measurements difficult to achieve. Certification of all involved professionals by accredited institutions and vascular labs is essential in minimizing variability. Vascular labs should always report interobserver and intraobserver variability alongside their results.

The task of AAA surveillance is complicated by the lack of standardization in measurement criteria. Conventional duplex US is documented as accurately measuring AAA diameter to within 3 mm of surgical specimens (13). When compared to CT, US consistently reports smaller measurements (48–51). Additional confusion stems from a lack of standardization of measurement criteria, with outer to outer wall, outer to inner wall, and inner to inner wall measurements all having been used (52). Outer to outer wall measurements are the ones more closely correlated with CT findings (52). The discrepancy seems to be resolved with 3D CEUS, as numerous studies have demonstrated strong correlation with CTA measurements (9, 36, 43, 48, 52).

Along the same lines, as previously reported, it appears clear that for the foreseeable future, post-EVAR surveillance will involve a multi-modality approach to graft imaging (42, 53, 54). As CTA is increasingly supplemented or replaced by US modalities, use of an accredited vascular lab with experienced technologists and internal quality controls allows for validation and concordance between measurements.

## Conclusion

Ultrasound is a cheap, accessible, and accurate screening tool for the previously undetected AAA, and is the imaging modality of choice in this setting. Novel technologies, including new ways of assessing wall strain and motion abnormalities, while not yet validated, have the future potential to allow for refinement of the rupture risk assessment beyond a simple size or ratio-based criteria. Future protocols may be refined to reflect local aortic wall motion and strain abnormalities for both development and rupture risk of AAA.

With the adoption of CEUS and 3D systems capable of spatial resolution, concurrent multiplanar data acquisition and reconstruction in transverse, coronal, and sagittal planes, US use is likely to increase in the pre-operative assessment, intraoperative use, and postoperative EVAR surveillance. Sensitivity and specificity of CEUS now match that of the traditional gold standards CTA and MRA in the post-EVAR evaluation of the aortic sac and endoleak presence. While not all patients will be candidates due to the limitations inherent in US technology, those who are will benefit from a decreased exposure to radiation and nephrotoxic contrast.

Ultrasound technology has the potential to supplement and potentially supplant CTA in many protocols, as recommendations are updated to reflect its evolving capabilities. As US tools evolve, we will continue to see a shift toward greater integration with, and replacement of, existing imaging modalities in the management of AAA.

## Author Contributions

MS and TG: data collection and writing of the manuscript. GA-K and RC: writing of the manuscript and critical revision. EA: writing of the manuscript, critical revision, and overall responsibility.

## Conflict of Interest Statement

The authors declare that the research was conducted in the absence of any commercial or financial relationships that could be construed as a potential conflict of interest.

## References

[1] Budd JS Finch DR Carter PG. A study of the mortality from ruptured abdominal aortic aneurysms in a district community. Eur J Vasc Surg (1989) 3(4):351–4. 10.1016/S0950-821X(89)80073-8 2788585

[2] Xu J Murphy SL Kochanek KD Bastian BA Deaths: Final Data for 2013. National Vital Statistics Reports. (Vol. 64). Hyattsville, MD: National Center for Health Statistics (2016). 26905861

[3] UK Statistics Authority. Deaths Registered in England and Wales, United Kingdom 2014. Kew; Richmond; Surrey: Office for National Statistics; The National Archives (2015).

[4] Kalef-Ezra JA Karavasilis S Ziogas D Dristiliaris D Michalis LK Matsagas M Radiation burden of patients undergoing endo-vascular abdominal aortic aneurysm repair. J Vasc Surg (2009) 49(2):283–7. 10.1016/j.jvs.2008.09.003 19216946

[5] Howells P Eaton R Patel AS Taylor P Modarai B. Risk of radiation exposure during endovascular aortic repair. Eur J Vasc Endovasc Surg (2012) 43(4):393–7. 10.1016/j.ejvs.2011.12.031 22265883

[6] Hertault A Maurel B Pontana F Martin-Gonzales T Spear R Sobocinski J Benefits of completion 3D angiography associated with contrast enhanced ultrasound to assess technical success after EVAR. Eur J Vasc Surg (2015) 49(5):541–8. 10.1016/j.ejvs.2015.01.010 25752417

[7] Rundback JH Nahl D Yoo V. Contrast-induced nephropathy. J Vasc Surg (2011) 54(2):575–9. 10.1016/j.jvs.2011.04.047 21741789

[8] Buijs RV Willems TP Tio RA Boersma HH Tielliu IF Slart RH Current state of experimental imaging modalities for risk assessment of abdominal aortic aneurysm. J Vasc Surg (2013) 57(3):851–9. 10.1016/j.jvs.2012.10.097 23357517

[9] Gargiulo M Gallito E Serra C Freyrie A Mascoli C Bianchini Massoni C Could four-dimensional contrast-enhanced ultrasound replace computed tomography angiography during follow up of fenestrated endografts? Results of a preliminary experience. Eur J Vasc Endovasc Surg (2014) 48(5):536–42. 10.1016/j.ejvs.2014.05.025 25023904

[10] Kok AM Nguyen VL Speelman L Brands PJ Schurink GW van de Vosse FN Feasibility of wall stress analysis of abdominal aortic aneurysms using three-dimensional ultrasound. J Vasc Surg (2015) 61(5):1175–84. 10.1016/j.jvs.2014.12.043 25701496

[11] Ormesher DC Lowe C Sedgwick N McCollum CN Ghosh J. Use of three-dimensional contrast-enhanced duplex ultrasound imaging during endovascular aneurysm repair. J Vasc Surg (2014) 60(6):1468–72. 10.1016/j.jvs.2014.08.095 25282700

[12] LaRoy LL Cormier PJ Matalon TAS Patel SK Turner DA Silver B. Imaging of abdominal aortic aneurysms. AJR Am J Roentgenol (1989) 152(4):785–92. 10.2214/ajr.152.4.785 2646870

[13] Cantisani V Ricci P Grazhdani H Napoli A Fanelli F Catalano C Prospective comparative analysis of colour-Doppler ultrasound, contrast-enhanced ultrasound, computed tomography and magnetic resonance in detecting endoleak after endovascular abdominal aortic aneurysm repair. Eur J Vasc Endovasc Surg (2011) 41(2):186–92. 10.1016/j.ejvs.2010.10.003 21095141

[14] Karatolios K Wittek A Ing D Nwe TH Bihari P Shelke A Method for aortic wall strain measurement with three-dimensional ultrasound speckle tracking and fitted finite element analysis. Ann Thorac Surg (2013) 96(5):1664–71. 10.1016/j.athoracsur.2013.06.037 23998405

[15] Derwich W Wittek A Pfister K Nelson K Bereiter-Hahn J Fritzen CP High resolution strain analysis comparing aorta and abdominal aortic aneurysm with real time three dimensional speckle tracking ultrasound. Eur J Vasc Endovasc Surg (2016) 51(2):187–93. 10.1016/j.ejvs.2015.07.042 26391962

[16] Guirguis-Blake JM Beil TL Senger CA Whitlock EP. Ultrasonography screening for abdominal aortic aneurysms: a systematic evidence review for the US Preventive Services Task Force. Ann Intern Med (2014) 160(5):321–9. 10.7326/M13-1844 24473919

[17] Bird AN Davis AM Screening for abdominal aortic aneurysm. JAMA (2015) 313(11):1156–7. 10.1001/jama.2015.0996 25781445

[18] Ellis M Powell JT Greenhalgh RM Limitations of ultrasonography for the surveillance of abdominal aortic aneurysms. Br J Surg (1991) 78(5):614–6. 10.1002/bjs.1800780529 2059819

[19] The UK Small Aneurysm Trial Participants. Mortality results for randomised controlled trial of early elective surgery or ultrasonographic surveillance for small abdominal aortic aneurysms. Lancet (1998) 352(9141):1649–55. 10.1016/S0140-6736(98)10137-X 9853436

[20] Ashton HA Buxton MJ Day NE Kim LG Marteau TM Scott RA The Multicentre Aneurysm Screening Study (MASS) into the effect of abdominal aortic aneurysm screening on mortality in men: a randomised controlled trial. Lancet (2002) 360(9345):1531–9. 10.1016/S0140-6736(02)11522-4 12443589

[21] Bredah K Sandholt B Lönn L Rouet L Ardon R Eiberg JP Three-dimensional ultrasound evaluation of small asymptomatic abdominal aortic aneurysms. Eur J Vasc Endovasc Surg (2015) 49:289–96. 10.1016/j.ejvs.2014.12.022 25662155

[22] Guirguis-Blake JM Beil TL Sun X Senger CA Whitlock EP Primary Care Screening for Abdominal Aortic Aneurysm: A Systematic Evidence Review for the US Preventive Services Task Force. [Internet]. Rockville, MD: Agency for Healthcare Research and Quality (2014). p. 1–145. 24555205

[23] LeFevre ML on Behalf of US Preventive Services Task Force. Screening for abdominal aortic aneurysm: US preventive services task force recommendation statement. Ann Intern Med (2014) 151(4):281–90. 10.7326/M14-1204 24957320

[24] Hirsh AT Haskal ZJ Hertzer NR Bakal CW Creager MA Halperin JL ACC/AHA 2005 guidelines for the management of patients with peripheral artery disease (lower extremity, renal, mesenteric, and abdominal aortic). J Am Coll Cardiol (2006) 47(6):1239–312. 10.1016/j.jacc.2005.10.009 16545667

[25] Mastracci TM Cinà CS and Canadian Society of Vascular Surgery Screening for abdominal aortic aneurysm in Canada: review and position statement of the Canadian Society for Vascular Surgery. J Vasc Surg (2007) 45(6):1268–76. 10.1016/j.jvs.2007.02.041 17543696

[26] Lindsay T on Behalf of Canadian Cardiovascular Society. Aortic Aneurysms Incidence, Screening and Indications for Repair [Internet]. CCS 2005 Peripheral Arterial Disease Consensus Document. Canadian Cardiovascular Society (2005). Available from: www.ccs.ca

[27] Chaikof EL Brewster DC Dalman RL Makaroun MS Illig KA Sicard GA The care of patients with an abdominal aortic aneurysm: the Society for Vascular Surgery practice guidelines. J Vasc Surg (2009) 50(4 Suppl):S2–49. 10.1016/j.jvs.2009.07.001 19786250

[28] Moll FL Powell JT Fraedrich G Verzini F Haulon S Waltham M Management of abdominal aortic aneurysms clinical practice guidelines of the European Society for Vascular Surgery. Eur J Vasc Endovasc Surg (2011) 41(Suppl1):S1–258. 10.1016/j.ejvs.2010.09.011 21215940

[29] van Disseldorp EMJ Hobelman KH Petterson NJ van de Vosse FN van Sambeek MR Lopata RG. Influence of limited field-of-view on wall stress analysis in abdominal aortic aneurysms. J Biomech (2016). 10.1016/j.jbiomech.2016.01.020 26924662

[30] Liu L Zheng H Williams L Zhang F Wang R Hertzberg J Development of a custom-designed echo particle image velocimetry system for multi-component hemodynamic measurement: system characterization and initial experimental results. Phys Med Biol (2008) 53(5):1397–412. 10.1088/0031-9155/53/5/015 18296769

[31] Zhang F Lanning G Mazzaro L Barker AJ Gates PE Strain WD In vitro and preliminary in vivo validation of echo particle image velocimetry in carotid vascular imaging. Ultrasound Med Biol (2011) 37(3):450–64. 10.1016/j.ultrasmedbio.2010.11.017 21316562 PMC3449315

[32] Huang SG Woo K Moos JM Han S Lew WK Chao A A prospective study of carbon dioxide digital subtraction versus standard contrast arteriography in the detection of endoleaks in endovascular abdominal aortic aneurysm repairs. Ann Vasc Surg (2013) 27(1):38–44. 10.1016/j.avsg.2012.10.001 23257072

[33] Kopp R Zürn W Weidenhagen R Meimarakis G Clevert DA First experience using intra-operative contrast enhanced ultrasound during endovascular aneurysm repair for infrarenal aortic aneurysms. J Vasc Surg (2010) 51(5):1103–10. 10.1016/j.jvs.2009.12.050 20420978

[34] Avgerinos ED Chaer RA Makaroun MS. Type II endoleaks. J Vasc Surg (2014) 60(5):1386–91. 10.1016/j.jvs.2014.07.100 25175637

[35] Gray C Goodman P Herron CC Lawler LP O’Malley MK O’Donohoe MK Use of colour duplex ultrasound as a first line surveillance tool following EVAR is associated with reduction in cost without compromising accuracy. Eur J Vasc Endovasc Surg (2012) 44(2):145–50. 10.1016/j.ejvs.2012.05.008 22717670

[36] Mirza TA Karthikesalingam A Jackson D Walsh SR Holt PJ Hayes P Duplex ultrasound and contrast-enhanced ultrasound versus computed tomography for the detection of endoleak after EVAR: systematic review and bivariate meta-analysis. Eur J Vasc Endovasc Surg (2014) 39(4):418–28. 10.1016/j.ejvs.2010.01.001 20122853

[37] Karthikesalingam A Al-Jundi W Jackson D Boyle JR Beard JD Holt PJE Systematic review and meta-analysis of duplex ultrasonography, contrast-enhanced ultrasonography or computed tomography for surveillance after endovascular aneurysm repair. Br J Surg (2012) 99(11):1514–23. 10.1002/bjs.8873 23001681

[38] Harrison GJ Oshin OA Vallabhaneni SR Brennan JA Fisher RK McWilliams RG. Surveillance after EVAR based on duplex ultrasound and abdominal radiography. Eur J Vasc Endovasc Surg (2011) 42(2):187–92. 10.1016/j.ejvs.2011.03.027 21546278

[39] Chaer RA Gushchin A Rhee R Marone L Cho JS Leers S Duplex ultrasound as the sole long-term surveillance method post-endovascular aneurysm repair: a safe alternative for stable aneurysms. J Vasc Surg (2009) 49(4):845–9. 10.1016/j.jvs.2008.10.073 19341877

[40] Beeman BR Doctor LM Doerr K McAfee-Bennett S Dougherty MJ Calligaro KD. Duplex ultrasound imaging alone is sufficient for midterm endovascular aneurysm repair surveillance: a cost analysis study and prospective comparison with computed tomography scan. J Vasc Surg (2009) 50(5):1019–24. 10.1016/j.jvs.2009.06.019 19656651

[41] Jung EM Rennert J Fellner C Uller W Jung W Schreyer A Detection and characterization of endoleaks following endovascular treatment of abdominal aortic aneurysms using contrast harmonic imaging (CHI) with quantitative perfusion analysis (TIC) compared with CT angiography. Ultraschall Med (2010) 31(6):564–70. 10.1055/s-0028-1109811 19941253

[42] Perini P Sediri I Midulla M Delsart P Mouton S Gautier C Single-centre prospective comparison between contrast-enhanced ultrasound and computed tomography angiography after EVAR. Eur J Vasc Endovasc Surg (2011) 42(6):797–802. 10.1016/j.ejvs.2011.09.003 21962588

[43] Motta R Rubaltelli L Vezzaro R Vida V Marchesi P Stramare R Role of multidetector CT angiography and contrast-enhanced ultrasound in redefining follow-up protocols after endovascular abdominal aortic aneurysm repair. Radiol Med (2012) 117(6):1079–92. 10.1007/s11547-012-0809-x 22430681

[44] Gilabert R Buñesch L Rael MI Gacía-Criado Á Burrel M Ayuso JR Evaluation of abdominal aortic aneurysm after endovascular repair: prospective validation of contrast-enhanced US with a second-generation US contrast agent. Radiology (2012) 264(1):269–77. 10.1148/radiol.12111528 22589321

[45] Bosch JAT Rouwet EV Peters CTH Jansen L Verhagen HJM Prins MH Contrast-enhanced ultrasound versus computed tomographic angiography for surveillance of endovascular abdominal aortic aneurysm repair. J Vasc Interv Radiol (2010) 21(5):638–43. 10.1016/j.jvir.2010.01.032 20363153

[46] Millen A Canavati R Harrison G McWilliams RG Wallace S Vallabhaneni SR Defining a role for contrast-enhanced ultrasound in endovascular aneurysm repair surveillance. J Vasc Surg (2013) 58(1):18–23. 10.1016/j.jvs.2012.12.057 23490295

[47] Partovi S Kaspar M Aschwanden M Lopresti C Madan S Uthoff H Contrast-enhanced ultrasound after endovascular aortic repair – current status and future perspectives. Cardiovasc Diagn Ther (2015) 5(6):454–63. 10.3978/j.issn.2223-3652.2015.09.04 26673398 PMC4666699

[48] Bredahl K Sandholt B Lönn L Rouet L Ardon R Eiberg JP Three-dimensional ultrasound evaluation of small asymptomatic abdominal aortic aneurysms. Eur J Vasc Endovasc Surg (2015) 49(3):289–96. 10.1016/j.ejvs.2014.12.022 25662155

[49] Manning BJ Kristmundsson T Sonesson B Resch T. Abdominal aortic aneurysm diameter: a comparison of ultrasound measurements with those from standard and three-dimensional computed tomography reconstruction. J Vasc Surg (2009) 50(2):263–8. 10.1016/j.jvs.2009.02.243 19631858

[50] Sprouse LR II Meier GH III Lesar CJ Demasi RJ Sood J Parent FN Comparison of abdominal aortic aneurysm diameter measurements obtained with ultrasound and computed tomography: is there a difference? J Vasc Surg (2003) 38(3):466–71. 10.1016/S0741-5214(03)00367-7 12947257

[51] Chiu KW Ling L Tripathi V Ahmed M Shrivastava V. Ultrasound measurement for abdominal aortic aneurysm screening: a direct comparison of the three leading methods. Eur J Vasc Endovasc Surg (2014) 47(4):367–73. 10.1016/j.ejvs.2013.12.026 24491283

[52] Leotta DF Paun M Beach KW Kohler TR Zierler RE Strandness DE Jr. Measurement of abdominal aortic aneurysms with three-dimensional ultrasound imaging: preliminary report. J Vasc Surg (2001) 33(4):700–7. 10.1067/mva.2001.112812 11296320

[53] Abbas A Hansrani V Sedwick N Ghosh J McCollum CN 3D contrast enhanced ultrasound for detection endoleak following endovascular aneurysm repair (EVAR). Eur J Vasc Endovasc Surg (2014) 47(5):487–92. 10.1016/j.ejvs.2014.02.002 24618331

[54] Kranokpiraksa P Kaufman JA. Follow-up of endovascular aneurysm repair: plain radiography, ultrasound, CT/CT angiography, MR imaging/MR angiography, or what? J Vasc Interv Radiol (2008) 19(6 Suppl):S27–36. 10.1016/j.jvir.2008.03.009 18502384

